# A comparison of nephrotoxicity between patients with a solitary-functioning kidney and those with bilateral-functioning kidneys in cisplatin-based chemotherapy for advanced urothelial carcinoma: a Japanese retrospective multi-institutional study

**DOI:** 10.1186/s12885-018-4186-z

**Published:** 2018-03-14

**Authors:** Takamitsu Inoue, Jun Miyazaki, Daishi Ichioka, Shintaro Narita, Susumu Kageyama, Mikio Sugimoto, Koji Mitsuzuka, Yusuke Shiraishi, Hidefumi Kinoshita, Hironobu Wakeda, Takeshi Nomoto, Eiji Kikuchi, Yoshiyuki Matsui, Keiko Fujie, Tomonori Habuchi, Hiroyuki Nishiyama

**Affiliations:** 1 Department of Urology, Akita University Graduate School of Medicine, Akita, 010-8543 Japan; 2 Department of Urology, Faculty of Medicine, University of Tsukuba, 1-1-1, Tennodai, Tsukuba, Ibaraki 305‑8575 Japan; 3 Department of Urology, Shiga University of Medical Science, Otsu, Shiga 520-2192 Japan; 4 Department of Urology, Kagawa University, Faculty of Medicine, Takamatsu, Kagawa 761-0701 Japan; 5 Department of Urology, Tohoku University Graduate School of Medicine, Sendai, Miyagi 980-8575 Japan; 6 Department of Urology, Hyogo College of Medicine, Nishinomiya, Hyogo 663-8501 Japan; 7 Department of Urology and Andrology, Kansai Medical University, Hirakata, Osaka 573-1191 Japan; 8 Department of Urology, Faculty of Medicine, University of Miyazaki, Miyazaki, 889-1692 Japan; 9 Department of Urology, Tokai University School of Medicine, Sagamihara, Kanagawa 259-1193 Japan; 10 Department of Urology, Keio University School of Medicine, Tokyo, 160-8582 Japan; 11 Department of Urology, Kyoto University Graduate School of Medicine, Kyoto, 606-8501 Japan; 12 Faculty of Medicine, University of Tsukuba, Tsukuba, Ibaraki 305-8575 Japan; 13 Tsukuba Clinical Research & Development Organization, University of Tsukuba, Tsukuba, Ibaraki 305-8575 Japan

**Keywords:** Urothelial carcinoma, Cisplatin, Nephrotoxicity, Nephroureterectomy, Solitary kidney

## Abstract

**Background:**

To compare the prevalence of nephrotoxicity between patients with a solitary-functioning kidney versus those with bilateral-functioning kidneys during the administration of cisplatin-based chemotherapy for advanced urothelial carcinoma.

**Methods:**

We retrospectively analyzed 244 advanced urothelial carcinoma patients treated with cisplatin-based chemotherapy between 2004 and 2010 at 17 institutes in Japan. The 24 h creatinine clearance, Cockcroft–Gault formula, and estimated glomerular filtration rate equation (eGFR), were compared before all chemotherapies. The urinary tract function status was determined based on the data of nephroureterectomy, hydronephrosis, and relief of upper urinary tract obstruction. A total of 244 patients were divided into four groups according to their urinary tract functioning status and eGFR results, including bilateral-functioning kidneys with pretreatment eGFR ≥60 mL/min/1.73 m^2^ group (*n* = 83, 34.0%); a solitary-functioning kidney with pretreatment eGFR ≥60 mL/min/1.73 m^2^ group (*n* = 36, 14.8%); bilateral-functioning kidneys with pretreatment eGFR < 60 mL/min/1.73 m^2^ group (*n* = 45, 18.4%); and a solitary-functioning kidney with pretreatment eGFR < 60 mL/min/1.73 m^2^ group (*n* = 80, 32.8%).

**Results:**

The prevalence of nephrotoxicity with impaired eGFR of > 10% and 30% from baseline in the post-third-course of chemotherapy was significantly higher in patients with bilateral-functioning kidneys than in those with a solitary-functioning kidney, among patients with pretreatment eGFR < 60 mL/min/1.73 m^2^ (*p* = 0.023 and *p* = 0.026). During all courses of chemotherapy, the prevalence of nephrotoxicity with impaired eGFR of > 20% from baseline were significantly higher in patients with bilateral-functioning kidneys than those with a solitary-functioning kidney among patients with pretreatment eGFR < 60 mL/min/1.73 m^2^ (*p* = 0.034), whereas no significant difference was observed among patients with pretreatment eGFR ≥60 mL/min/1.73 m^2^.

**Conclusions:**

The results suggest that cisplatin-based chemotherapy may have more nephrotoxicity in patients with bilateral-functioning kidneys than in those with a solitary-functioning kidney.

**Electronic supplementary material:**

The online version of this article (10.1186/s12885-018-4186-z) contains supplementary material, which is available to authorized users.

## Background

Cisplatin-based combination chemotherapies have been the standard regimen for patients with advanced urothelial carcinoma (UC) since the approval of cisplatin in the United States in 1993 [1]. The standard regimens for patients with advanced UC are methotrexate, vinblastine, doxorubicin, and cisplatin (MVAC), or gemcitabine and cisplatin (GC). The median overall survival of these two regimens is 13.8 and 14.8 months, respectively [2, 3]. Although cisplatin is a key drug for the treatment of patients with advanced UC, a significant nephrotoxicity associated with cisplatin therapy restricts its use to patients with appropriate kidney function [4].

To estimate the suitability of cisplatin treatment before the initiation of therapy, the Cockcroft–Gault formula (C-G), a modification of diet in renal disease (MDRD) formula, and/or a 24 h creatinine clearance test (24hCCr) have been widely used to estimate a glomerular filtration rate (GFR) [5]. A patient with a GFR < 60 mL/min is usually defined as having the chronic renal disease (CKD) and cisplatin-ineligible [4]. For cisplatin-ineligible patients, carboplatin-based combination chemotherapies have been the most favored regimens, using Calvert’s formula to adjust the dose of carboplatin according to the estimated GFR. Outcomes for cisplatin-eligible advanced UC patients treated with carboplatin-based chemotherapies, such as gemcitabine plus carboplatin, with a median overall survival of 9.0 months, were poorer than those for patients treated with cisplatin-based chemotherapies [6, 7]. However, in cisplatin-ineligible advanced UC patients, the median overall survival of patients treated with carboplatin-based combination chemotherapies are reported 7.2-16.3 months [8–11], which is almost similar (i.e., around 10 months) in those treated with cisplatin-based chemotherapies, including the reduction of cisplatin [12, 13] or a split dose of cisplatin regimens [14–16].

Following radical nephroureterectomy, approximately 78–81% of patients with upper tract urothelial carcinoma (UTUC) are cisplatin-ineligible (with eGFR < 60 mL/min/1.73 m^2^) [17, 18]. Therefore, the recommended treatment for patients with advanced UTUC, listed in the clinical guidelines (e.g., the 2015 European Association of Urology Guidelines), is neoadjuvant chemotherapy only, with consideration made for the fact that chemotherapy-related nephrotoxicity from platinum derivatives may significantly reduce survival [9, 19]. However, Lene et al. recently showed that renal cell carcinoma patients with surgically-induced CKD (CKD-S) have a relatively low risk of progressive renal function decline, whereas those with medically-induced CKD (CKD-M) have an increased risk [20]. In addition, a previous report from Korea showed that cisplatin-based chemotherapy was safe in the majority of patients who underwent nephroureterectomy [21]. It is plausible that the CKD-S patients, who underwent nephroureterectomy or who have ipsilateral hydronephrosis and an intact solitary-functioning kidney without medical comorbidities, have some potential endurance for nephrotoxicity in cisplatin-based chemotherapy. However, currently there is an insufficient amount of data to support recommendations of chemotherapy regimens for CKD-S advanced UC patients with a solitary-functioning kidney [5, 19].

In this study, the prevalence of nephrotoxicity in cisplatin-based chemotherapy for advanced UC was retrospectively compared in cisplatin-ineligible patients with a solitary-functioning kidney versus those with bilateral-functioning kidneys, using a Japanese multi-institutional database.

## Methods

In this study, we retrospectively evaluated 345 advanced or unresectable UC patients who underwent systemic chemotherapy between 2004 and 2010 at 17 institutes in Japan (CURE study group). Patients who underwent neo-adjuvant chemotherapy or chemoradiation for bladder preservation were excluded from this study. All cases required pathological confirmation of UC, except for patients with upper urinary tract cancer, who were instead diagnosed based on positive urinary cytology and radiological examinations. The concept of the study was approved by the internal ethical committees at all of the 17 institutions involved. Informed consent for chemotherapies was obtained from all the patients. Informed consent to participate in the study was not obtained with an opt-out statement on the website of all of the 17 institutions involved. Follow-up data were acquired in December 2013. All data were collected from medical records at each institution and registered by a secretariat server on the website.

We selected 244 patients, who underwent cisplatin-based combination regimens as a first-line chemotherapy and had data on their kidney function status. The cisplatin-based combination chemotherapies included in this study were GC (*n* = 103, 42.2%); MVAC (*n* = 98, 40.2%); methotrexate, epirubicin, and cisplatin (MEC) (*n* = 35, 14.3%); and gemcitabine, cisplatin, and docetaxel (GCD) (*n* = 8, 3.3%). The selection of chemotherapy regimens was based on the preference of each institute.

The urinary tract function status was evaluated and defined by the following criteria: 1) a patient who underwent radical nephroureterectomy was defined as having a contralateral solitary-functioning kidney; 2) a patient who had hydronephrosis (regardless of grade) was defined as having an ipsilateral non-functional kidney; and 3) a patient who underwent a relief of upper urinary tract obstruction, including placement of an internal ureteral stent or nephrostomy, was defined as having a functional ipsilateral kidney. Patients without information on The urinary tract function status were excluded from this study. The 24hCCr, C-G, and the Japanese estimated GFR equation, which originated from the MDRD equation recommended by the Japanese Society of Nephrology (eGFR) [22], were compared for GFR estimation before chemotherapies. The criterion of pretreatment eGFR ≥60 mL/min/1.73 m^2^ was used to define cisplatin eligibility.

The decision to reduce the dose was made by the physician who treated each patient. Only dose reductions of cisplatin were evaluated in this study; however, data on skipped doses were included for all agents. Serum creatinine levels and eGFR, measured during chemotherapy, were included for the morning of day one (pre-), the day of the maximum level of creatinine (max-), and day 22 (post-) until the fourth course. The data after the fifth course of first-line cisplatin-based chemotherapy was not evaluated in this study. Investigators reported the observed data, including the values of serum creatinine levels, into the website system following first-line chemotherapy, retrospectively.

The chi-square test was used to compare the proportions of clinical parameters between patients with the bilateral and/or the solitary-functioning kidney. A one-way repeated measures analysis of variance (ANOVA)-covariance model and Student’s t-test were used to determine the between-group differences and the within-group changes over time, respectively. All statistical analyses were performed using SPSS software, version 22.0 (IBM Corp., Armonk, NY, USA), and *P*-values of < 0.05 were considered significant.

## Results

### Determination of the urinary tract function status

According to the criteria described above for the urinary tract function status, regarding nephroureterectomy, hydronephrosis, and relief of the upper urinary tract obstruction, 128 (52.5%) and 116 (47.5%) patients were defined as having bilateral and solitary-functioning kidneys, respectively. Nephroureterectomy and relief of upper urinary tract obstruction, including the placement of an internal ureteral stent or nephrostomy, was performed in 49 (20.0%) and 60 (24.5%) patients, respectively. Hydronephroses were left untreated prior to the initiation of chemotherapy in 16 (6.6%) patients. The urinary tract function status of all the 244 patients is listed in Table 1.

**Table 1:** Determination of the urinary tract functioning status and pretreatment eGFR of the 244 patients who underwent cisplatin-based chemotherapies

	Pretreatment eGFR ≥60 mL/min/1.73m^2^		Pretreatment eGFR < 60 mL/min/1.73m^2^	
	Bilateral functioning kidneys (*n* = 83)	Solitary functioning kidney (*n* = 36)	Bilateral functioning kidneys (*n* = 45)	Solitary functioning kidney (*n* = 80)
Bilateral functoinal kidneys status				
BC with bilateral intact kidneys	63 (75.9%)	–	23 (51.2%)	–
BC with an unilateral nephrostomy	4 (4.8%)	–	9 (20.0%)	–
UTUC without nephroureterectomy or hydronephrosis	11 (13.3%)	–	11 (24.4%)	–
UTUC with an ipsilateral nephrostomy	2 (2.4%)	–	1 (2.2%)	–
BC + UTUC without nephroureterectomy or hydronephrosis	3 (3.6%)	–	1 (2.2%)	–
Solitary functional kidney status				
UTUC after nephroureterectomy	–	7 (19.4%)	–	37 (46.4%)
UTUC without nephroureterectomy with an ipsilateral	–	11 (30.5%)	–	22 (27.5%)
UTUC in the solitary kidney without hydronephroisis	–	1 (2.7%)	–	2 (2.5%)
UTUC in the solitary kidney with the ipsilateral nephrostomy	–	0 (0.0%)	–	1 (1.2%)
BC with an unilateral hydroneohrosis	–	7 (19.4%)	–	5 (6.2%)
BC with bilateral hydronephroses with unilateral nephrostomy	–	2 (5.6%)	–	8 (10.0%)
BC with a solitary kidney	–	3 (8.4%)	–	1 (1.2%)
BC + UTUC after an unilateral nephroureterectomy	–	3 (8.4%)	–	2 (2.5%)
BC + UTUC with an unilateral hydronephrosis	–	2 (5.6%)	–	2 (2.5%)

*BC* bladder cancer, *UTUC* upper urinary tract urothelial cancer

### Comparison of the methods to estimate GFR

**Fig. 1:**
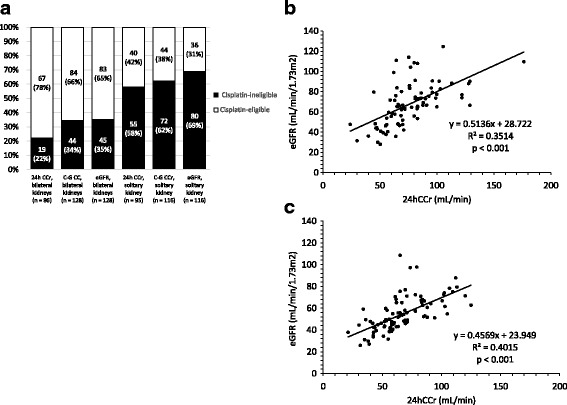
Comparison of the GFR estimation between 24 hCCr, C-G CCr, eGFR, and urinary tract function status. **a**: Proportions of cisplatin-eligible and ineligible patients based on the methods used to estimate the GFR. 24hCCr estimation was performed in 188 (77.0%) patients before chemotherapy treatment. The proportion of cisplatin-ineligible patients based on eGFR results was significantly higher than when using 24hCCr using the threshold of 60 mL/min (p = 0.040). The proportion of cisplatin-ineligible patients with a solitary-functioning kidney was significantly higher than those with bilateral-functioning kidneys using both 24hCCr and eGFR tests (*p* < 0.010 and *p* < 0.010). **b**: 24hCCr and eGFR were significantly correlated in patients with bilateral-functioning kidneys (r^2^ = 0.351, *p* < 0.001). **c**: 24hCCr and eGFR were significantly correlated in patients with a solitary-functioning kidney (r^2^ = 0.402, *p* < 0.001)

The estimated 24hCCr test was performed in 188 (77.0%) patients before initiation of chemotherapy. Using results from the 24hCCr test, 19 (22.1%) and 55 (57.9%) patients were diagnosed as cisplatin-ineligible with bilateral and solitary-functioning kidneys, respectively, while eGFR results defined 45 (35.2%) and 80 (69.0%) patients as cisplatin-ineligible with bilateral and solitary-functioning kidneys, respectively. The proportion of patients defined as cisplatin-ineligible was significantly higher when using eGFR results versus 24hCCr results (*p* = 0.040). Furthermore, the proportion of cisplatin-ineligible patients who had a solitary-functioning kidney was significantly higher than that of patients with bilateral-functioning kidneys, using both the 24hCCr and the eGFR tests (*p* <  0.010 and *p* <  0.010; Fig. 1a). The 24hCCr and eGFR tests were significantly correlated in patients with bilateral and solitary-functioning kidneys (r^2^ = 0.351, *p* < 0.001 and r^2^ = 0.402, *p* < 0.001; Fig. 1b, c, respectively).

A total of 244 patients were divided into four groups according to their urinary tract functioning status and eGFR results as listed in Table 1, including bilateral-functioning kidneys with pretreatment eGFR ≥60 mL/min/1.73 m^2^ group (*n* = 83, 34.0%); a solitary-functioning kidney with pretreatment eGFR ≥60 mL/min/1.73 m^2^ group (*n* = 36, 14.8%); bilateral-functioning kidneys with pretreatment eGFR < 60 mL/min/1.73 m^2^ group (*n* = 45, 18.4%); and a solitary-functioning kidney with pretreatment eGFR < 60 mL/min/1.73 m^2^ group (*n* = 80, 32.8%).

### Comparisons of the patient comorbidity, chemotherapy regimen, dose- reduction, and skipped administration

The demographic data of the four groups at the beginning of chemotherapy treatment is listed in Table 2. The proportion of patients < 70 years of age with a solitary-functioning kidney was significantly higher than that of those with bilateral-functioning kidneys, for all patients and for patients with pretreatment eGFR ≥60 mL/min/1.73 m^2^ (*p* = 0.016 and *p* = 0.039, respectively). The proportion of patients with a solitary-functioning kidney who had lung or liver metastasis was significantly higher than that of patients with bilateral-functioning kidneys with pretreatment eGFR ≥60 mL/min/1.73 m^2^ (both *p* = 0.018). The proportion of patients with bilateral-functioning kidneys and lymph node metastasis was significantly higher than patients with a solitary-functioning kidney with pretreatment eGFR < 60 mL/min/1.73 m^2^ (*p* = 0.010; Table 2). No significant differences were observed in the proportion of medical comorbidities between bilateral and solitary-functioning kidney patients with pretreatment eGFR ≥60 or < 60 mL/min/1.73 m^2^.

**Table 2:** Demographic data of analyzed 244 patients who underwent cisplatin-based chemotherapies

		All patients			Pretreatment eGFR ≥60 mL/min/1.73m^2^			Pretreatment eGFR < 60 mL/min/1.73m^2^		
		Pretreatment eGFR ≥60 mL/min/1.73m^2^ (*n* = 119)	Pretreatment eGFR < 60 mL/min/1.73m^2^ (*n* = 125)	p	Bilateral functioning kidneys (*n* = 83)	Solitary functioning kidney (*n* = 36)	p	Bilateral functioning kidneys (*n* = 45)	Solitary functioning kidney (*n* = 80)	p
Age	< 70	78 (65.5%)	63 (50.4%)	0.016	49 (58.9%)	29 (80.5%)	0.039	20 (44.4%)	43 (53.7%)	0.32
	≥70	41 (34.4%)	62 (49.6%)		34 (41.1%)	7 (19.5%)		25 (55.6%)	37 (46.3%)	
PS	0-1	111 (93.2%)	117 (93.6%)	0.91	77 (92.7%)	34 (94.4%)	0.94	42 (93.3%)	75 (93.8%)	0.083
	2-4	8 (6.7%)	8 (6.4%)		6 (7.3%)	2 (5.6%)		3 (6.7%)	5 (6.2%)	
Sex	Male	86 (72.3%)	87 (69.6%)	0.64	61 (73.5%)	25 (69.4%)	0.65	32 (71.1%)	55 (68.7%)	0.78
	Female	33 (27.7%)	38 (30.4%)		22 (26.5%)	11 (30.6%)		13 (28.9%)	25 (31.3%)	
Comorbidities	yes	15 (12.6%)	17 (14.2%)	0.81	13 (15.7%)	2 (5.6%)	0.12	8 (17.8%)	9 (11.2%)	0.36
	no / unknown	104 (87.4%)	108 (86.4%)		70 (84.3%)	34 (94.4%)		37 (82.2%)	71 (88.8%)	
	DM	3 (2.5%)	4 (3.2%)		2 (2.4%)	1 (2.7%)		2 (4.4%)	2 (2.4%)	
	Glomerulonephritis	1 (0.8%)	1 (0.8%)		1 (1.2%)	0 (0.0%)		0 (0.0%)	1 (1.2%)	
	Others	11 (9.2%)	12 (9.6%)		10 (12.1%)	1 (2.7%)		6 (13.3%)	6 (7.5%)	
Cancer location	BC	79 (66.5%)	46 (36.8%)	< 0.001	67 (80.7%)	12 (33.3%)	< 0.001	32 (71.1%)	14 (17.5%)	< 0.001
	UTUC	32 (26.8%)	74 (59.2%)		13 (15.7%)	19 (52.8%)		12 (26.7%)	62 (77.5%)	
	BC + UTUC	8 (6.7%)	5 (4.0%)		3 (3.6%)	5 (13.9%)		1 (2.2%)	4 (5.0%)	
Surgery	No surgery	85 (71.5%)	73 (58.4%)	0.033	63 (75.9%)	22 (61.1%)	< 0.001	38 (84.4%)	35 (43.7%)	< 0.001
	Cystectomy	25 (21.0%)	14 (11.2%)		20 (24.1%)	5 (13.9%)		7 (15.6%)	7 (8.8%)	
	Nephroureterectomy	7 (5.8%)	37 (29.6%)		0 (0.0%)	7 (19.4%)		0 (0.0%)	37 (46.3%)	
	Cystectomy + nephroureterectomy	2 (1.7%)	1 (0.8%)		0 (0.0%)	2 (5.6%)		0 (0.0%)	1 (1.2%)	
Metastatic site	Lymph node	77 (64.7%)	68 (54.4%)	0.10	55 (66.2%)	22 (61.1%)	0.58	33 (73.3%)	35 (43.8%)	0.001
	Lung	41 (34.5%)	45 (36.0%)	0.80	23 (12.1%)	18 (50.0%)	0.018	16 (35.6%)	29 (36.3%)	0.94
	Liver	24 (20.2%)	18 (14.4%)	0.23	12 (14.5%)	12 (33.3%)	0.018	6 (13.3%)	12 (15.0%)	0.79
	Bone	22 (18.5%)	17 (13.6%)	0.29	13 (15.7%)	9 (25.0%)	0.23	6 (13.3%)	11 (13.7%)	0.95
	Others	28 (23.5%)	32 (25.6%)	0.70	15 (18.1%)	13 (36.1%)	0.033	9 (20.0%)	23 (28.7%)	0.28

*BC* bladder cancer, *UTUC* upper urinary tract urothelial cancer

No significant differences were observed in the selection of chemotherapy regimens between patients with the bilateral and solitary-functioning kidneys with respect to those pretreatment eGFR ≥60 or < 60 mL/min/1.73 m^2^. The proportion of patients whose cisplatin dose was reduced was significantly higher in patients with pretreatment eGFR < 60 mL/min/1.73 m^2^ than in those with ≥60 mL/min/1.73 m^2^ (*p* < 0.001). No significant differences were observed during four courses of cisplatin-based chemotherapy with regards to the patients with cisplatin dose reduction or skipped administration of chemotherapy agents between patients with bilateral and solitary-functioning kidneys with respect to those with pretreatment eGFR ≥60 or < 60 mL/min/1.73 m^2^ (Table 3, Additional file 1: Figure S1).

**Table 3:** Comparison of the proportion of the selected chemotherapy regimens, dose-reduction, and skip administration

		All patients			Cisplatin-eligible (eGFR ≥60 mL/min/1.73m^2^)			Cisplatin-ineligible (eGFR < 60 mL/min/1.73 m2)		
		Cisplatin-eligible (*n* = 119)	Cisplatin-ineligible (*n* = 125)	p	Bilateral functioning kidneys (*n* = 83)	Solitary functioning kidney (*n* = 36)	p	Bilateral functioning kidneys (*n* = 45)	Solitary functioning kidney (*n* = 80)	p
Chemotherapy	GC	48 (40.3%)	55 (44.0%)	0.56	35 (42.2%)	13 (36.2%)	0.530	24 (53.3%)	31 (38.8%)	0.160
	MVAC	52 (43.6%)	46 (36.8%)	0.27	37 (44.6%)	15 (41.6%)	0.760	14 (31.1%)	32 (40.0%)	0.110
	MEC	16 (13.4%)	19 (15.2%)	0.70	9 (10.8%)	7 (19.4%)	0.210	4 (8.9%)	15 (18.7%)	0.140
	Gemcitabine + Cisplatin + Docetaxel	3 (2.5%)	5 (4.0%)	0.51	2 (2.4%)	1 (2.8%)	0.900	3 (6.7%)	2 (2.5%)	0.850
Cisplatin dose reduction										
all courses	Yes	10 (8.4%)	42 (33.6%)	< 0.001	6 (7.2%)	4 (11.1%)	0.480	16 (35.5%)	26 (32.5%)	0.730
1st course	Yes	9/119 (7.5%)	48/125 (38.4%)	< 0.001	6/83 (7.2%)	3/36 (8.3%)	0.834	16/45 (35.5%)	32/80 (39.9%)	0.623
	99-80%	8/119 (6.7%)	15/125 (12.0%)	0.158	5/83 (6.0%)	3/36 (8.3%)	0.644	5/45 (11.1%)	10/80 (12.5%)	0.810
	< 80%	1/119 (0.8%)	33/125 (26.4%)	< 0.001	1/83 (1.2%)	0/36 (0.0%)	0.508	11/45 (24.4%)	22/80 (27.5%)	0.709
2nd course	Yes	8/104 (7.7%)	39/110 (35.3%)	< 0.001	5/72 (8.3%)	3/32 (9.3%)	0.667	13/42 (30.8%)	26/68 (38.1%)	0.437
	99-80%	7/104 (6.7%)	9/110 (8.1%)	0.686	4/72 (6.9%)	3/32 (9.3%)	0.473	2/42 (4.7%)	7/68 (10.2%)	0.303
	< 80%	1/104 (1.0%)	30/110 (27.2%)	< 0.001	1/72 (1.4%)	0/32 (0.0%)	0.500	11/42 (26.1%)	19/68 (27.9%)	0.841
3rd course	Yes	4/81 (4.9%)	23/82 (28.0%)	< 0.001	2/54 (3.8%)	2/27 (7.4%)	0.468	6/24 (24.9%)	17/58 (29.2%)	0.690
	99-80%	3/81 (3.7%)	7/82 (8.5%)	0.198	1/54 (1.9%)	2/27 (7.4%)	0.212	1/24 (4.1%)	6/58 (10.3%)	0.362
	< 80%	1/81 (1.2%)	16/82 (19.5%)	< 0.001	1/54 (1.9%)	0/27 (0.0%)	0.476	5/24 (20.8%)	11/58 (18.9%)	0.846
4th course	Yes	2/59 (3.4%)	15/59 (25.4%)	< 0.001	0/39 (0.0%)	2/20 (10.0%)	0.044	4/20 (20.0%)	12/39 (30.7%)	0.378
	99-80%	2/59 (3.4%)	6/59 (10.2%)	0.142	0/39 (0.0%)	2/20 (10.0%)	0.044	1/20 (5.0%)	5/39 (12.8%)	0.346
	< 80%	0/59 (0.0%)	9/59 (15.2%)	< 0.001	0/39 (0.0%)	0/20 (0.0%)	1.000	3/20 (15.0%)	7/39 (17.9%)	0.775
Skip										
all courses	Yes	65 (54.6%)	70 (56.0%)	0.83	49 (59.0%)	16 (44.4%)	0.140	23 (51.1%)	47 (58.8%)	0.400
	No	54 (45.3%)	55 (44.0%)		34 (40.9%)	20 (55.6%)		22 (48.9%)	33 (41.2%)	
1st course	day 8	8 (6.7%)	15 (12.0%)	0.16	7 (8.4%)	1 (2.7%)	0.250	4 (8.9%)	11 (13.7%)	0.420
	day 15	54 (45.3%)	54 (43.2%)	0.73	40 (48.1%)	14 (38.9%)	0.340	17 (37.7%)	37 (46.3%)	0.350
	day 22	8 (6.7%)	13 (10.4%)	0.30	6 (7.2%)	2 (5.6%)	0.730	4 (4.4%)	9 (11.3%)	0.680

*BC* bladder cancer, *UTUC* upper urinary tract urothelial cancer

### Comparison of nephrotoxicity during cisplatin-based chemotherapy

**Fig. 2:**
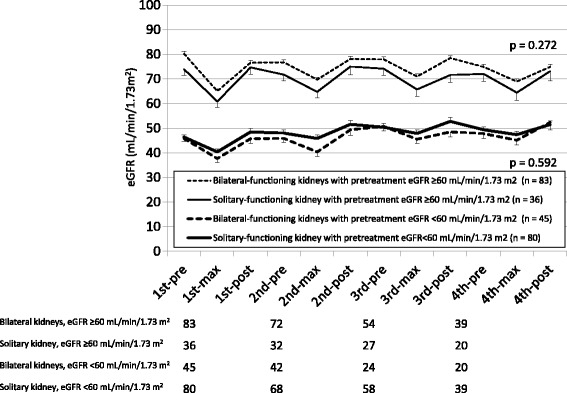
Comparison of the mean eGFR value during cisplatin-based chemotherapies between bilateral and solitary-functioning kidneys, in patients with eGFR ≥60 and < 60 mL/min/1.73 m^2^. No significant differences were observed in the mean eGFR between patients with bilateral and solitary-functioning kidneys

In the comparison of nephrotoxicity between the patients with bilateral- and solitary-functioning kidneys, no significant differences were observed in the mean eGFR between both patients with pretreatment eGFR ≥60 and < 60 mL/min/1.73 m^2^ (Fig. 2). However, the prevalence of nephrotoxicity with impaired eGFR of > 10% and 30% from baseline in the post-third-course of chemotherapy, with rates significantly higher in patients with bilateral-functioning kidneys than in those with a solitary-functioning kidney among patients with pretreatment eGFR < 60 mL/min/1.73 m^2^ (*p* = 0.023 and *p* = 0.026; Table 4, Additional file 2: Figure S2). During all courses of chemotherapy, the prevalence of nephrotoxicity with impaired eGFR of > 20% from baseline were significantly higher in patients with bilateral-functioning kidneys than in those with a solitary-functioning kidney among patients with pretreatment eGFR < 60 mL/min/1.73 m^2^ (*p* = 0.034; Table 4), whereas no significant difference was observed among patients with pretreatment eGFR ≥60 mL/min/1.73 m^2^.

**Table 4:** Comparison of nephrotoxicity during cisplatin-based chemotherapy

		All patients			Pretreatment eGFR ≥60 mL/min/1.73m^2^			Pretreatment eGFR < 60 mL/min/1.73m^2^		
Course	% Baseline eGFR impairment	Pretreatment eGFR ≥60 mL/min/1.73m^2^ (*n* = 119) impaird patient /n (%)	Pretreatment eGFR < 60 mL/min/1.73 m^2^ (*n* = 125) impaird patient /n (%)	p	Bilateral functioning kidneys (*n* = 83) impaird patient /n (%)	Solitary functioning kidney (*n* = 36) impaird patient /n (%)	p	Bilateral functioning kidneys (*n* = 45) impaird patient /n (%)	Solitary functioning kidney impaird patient /n (%)	p
post 1st course	> 10% impairment	38/119 (31.9%)	23/125 (18.4%)	0.015	28/83 (33.7%)	10/36 (27.7%)	0.52	9/45 (20.0%)	15/80 (18.7%)	0.73
	> 20% impairment	19/119 (15.9%)	10/125 (8.0%)	0.054	15/83 (18.0%)	4/36 (11.1%)	0.34	6/45 (13.3%)	5/80 (6.2%)	0.17
	> 30% impairment	4/119 (3.4%)	4/125 (3.2%)	0.94	3/83 (3.6%)	1/36 (2.7%)	0.81	3/45 (6.6%)	2/80 (2.5%)	0.098
post 2nd course	> 10% impairment	29/104 (27.8%)	17/110 (14.5%)	0.026	20/72 (27.7%)	9/32 (28.1%)	0.97	10/42 (23.8%)	7/68 (10.2%)	0.056
	> 20% impairment	13/104 (12.5%)	6/110 (5.4%)	0.070	9/72 (12.5%)	4/32 (12.5%)	1.00	4/42 (9.5%)	2/68 (2.9%)	0.14
	> 30% impairment	6/104 (5.8%)	3/110 (2.7%)	0.26	4/72 (5.5%)	2/32 (6.2%)	0.88	2/42 (4.7%)	1/68 (1.4%)	0.30
post 3rd course	> 10% impairment	29/78 (37.2%)	15/82 (18.3%)	0.011	17/54 (31.4%)	12/27 (44.4%)	0.25	8/24 (33.3%)	7/58 (12.1%)	0.023
	> 20% impairment	18/78 (23.0%)	7/82 (8.5%)	0.015	14/54 (25.9%)	4/27 (14.8%)	< 0.001	4/24 (16.6%)	3/58 (5.2%)	0.091
	> 30% impairment	5/78 (6.4%)	2/82 (2.4%)	0.23	2/54 (3.7%)	3/27 (11.1%)	0.19	2/24 (8.3%)	0/58 (0.0%)	0.026
post 4th course	> 10% impairment	26/59 (44.1%)	13/59 (22.0%)	0.010	17/39 (43.6%)	9/20 (45.0%)	0.91	4/20 (20.0%)	9/39 (23.0%)	0.78
	> 20% impairment	12/59 (20.3%)	3/59 (5.1%)	0.028	9/39 (23.0%)	3/20 (15.0%)	0.46	2/20 (10.0%)	1/39 (2.5%)	0.21
	> 30% impairment	7/59 (11.9%)	1/59 (1.7%)	0.012	5/39 (12.8%)	2/20 (10.0%)	0.75	1/20 (5.0%)	0/39 (0.0%)	0.16
during all courses	> 10% impairment	56/119 (47.1%)	32/125 (25.6%)	< 0.001	39/83 (46.9%)	17/36 (47.2%)	0.98	16/45 (33.3%)	18/80 (22.5%)	0.14
	> 20% impairment	25/119 (21.0%)	17/125 (13.6%)	0.125	18/83 (21.6%)	7/36 (19.4%)	0.78	10/45 (22.2%)	7/80 (8.7%)	0.034
	> 30% impairment	16/119 (13.4%)	7/125 (5.6%)	0.036	11/83 (13.2%)	5/36 (13.8%)	0.92	5/45 (11.1%)	3/80 (3.8%)	0.11

In the comparison of nephrotoxicity between patients with pretreatment eGFR ≥60 and < 60 mL/min/1.73 m^2^, the prevalence of impaired eGFR of > 10% from baseline in the post-first to fourth courses in patients with pretreatment eGFR ≥60 mL/min/1.73 m^2^ was significantly higher than those in patients with pretreatment eGFR < 60 mL/min/1.73 m^2^ (*p* = 0.015, *p* = 0.026, *p* = 0.011, and *p* = 0.010, respectively; Table 4). The prevalence of impaired eGFR of > 20% from baseline in the post-third and fourth courses in patients with pretreatment eGFR ≥60 mL/min/1.73 m^2^ was significantly higher than those in patients with pretreatment eGFR < 60 mL/min/1.73 m^2^ (*p* = 0.015 and *p* = 0.028; Table 4). Furthermore, the prevalence of impaired eGFR of > 30% from baseline at the post-fourth course in patients with pretreatment eGFR ≥60 mL/min/1.73 m^2^ was significantly higher than that of patients with pretreatment eGFR < 60 mL/min/1.73 m^2^ (*p* = 0.012; Table 4). During all courses of chemotherapy, the prevalence of impaired eGFR of > 10% and 30% from baseline in patients with pretreatment eGFR ≥60 mL/min/1.73 m^2^ was significantly higher than those in patients with pretreatment eGFR < 60 mL/min/1.73 m^2^ (*p* < 0.001 and *p* = 0.036; Table 4). No patients required hemodialysis.

## Discussion

In advanced UTUC patients following nephroureterectomy, the proportion of cisplatin-ineligible patients is reportedly 78–81%, and there is often a therapeutic dilemma in the chemotherapy for these patients [17]. In total, 37 of the 44 (78.7%) patients who underwent nephroureterectomy in this study were categorized into the group of eGFR < 60 mL/min/1.73 m^2^. Although carboplatin-based combination chemotherapies have been the most favored regimens in these kinds of patients, cisplatin-based chemotherapies were selected in this study at the discretion of individual institutes, likely because of the evidence of the better outcomes of cisplatin-based chemotherapies in cisplatin-eligible advanced UC [6]. However, the nephrotoxicity of cisplatin-based chemotherapy in patients with an intact solitary kidney has not been extensively investigated. The present study is the first retrospective study comparing the nephrotoxicity between bilateral- and solitary-functioning kidneys in CKD patients during cisplatin-based chemotherapies.

In the evaluation of the mean value of eGFR in this study, the kidney function did not deteriorate during four courses. Moreover, the mean kidney function between patients with bilateral- and solitary-functioning kidneys did not significantly differ in both patients with pretreatment eGFR ≥60 and < 60 mL/min/1.73 m^2^. However, there should be a significant bias noted in that the patients who did not continue the chemotherapy due to nephrotoxicity were excluded from the next course in the evaluation of the mean value of eGFR. Therefore, we focused more on the nephrotoxicity of individual patients; indeed, the nephrotoxicity was more frequently observed in patients with bilateral-functioning kidneys than in those with a solitary-functioning kidney in patients with pretreatment eGFR < 60 mL/min/1.73 m^2^. The details of cisplatin-based chemotherapy were almost not found to be significantly different between groups based on the selection of chemotherapy regimen, reduction of cisplatin dose and/or the requirement to skip administration of the chemotherapy agents. Although the difference was slight, our data suggest that kidney function is more likely to be injured by cisplatin-based chemotherapies in patients with bilateral-functioning kidneys than in those with a solitary-functioning kidney.

The reason for our results may be that the microstructures of the nephrons in CKD-S patients following contralateral nephroureterectomy or hydronephrosis are less deteriorated and more resistant to chemotherapy than those in CKD-M patients with bilateral-functioning kidneys. Although the difference was not significant, comorbidities were more frequently observed in patients with bilateral-functioning kidneys than in those with a solitary-functioning kidney. Histories of diabetes mellitus and cardiovascular disease were found to be significant risk factors to predict severe acute kidney injury induced by cisplatin-based chemotherapy in a previous evaluation of 1721 cancer patients [23]. Although the mean body mass index was not significantly different between the groups in this series, there is a possibility that the potential deterioration of the kidney such as smoking status, hypertension, or hyperuricemia might be more prevalent in patients with bilateral-functioning kidneys than in those with a solitary-functioning kidney.The differences of these factors were not obvious because of the retrospective study.

Another finding in this retrospective study was that patients with impaired kidney function were more frequently observed in patients with pretreatment eGFR ≥60 than in < 60 mL/min/1.73 m^2^, probably because of the frequent dose reductions in patients with pretreatment eGFR < 60 mL/min/1.73 m^2^. Moreover, the prevalence of the nephrotoxicity increased as chemotherapy courses progressed, during the four courses in patients with pretreatment eGFR ≥60 mL/min/1.73 m^2^, with an 8.4% reduction in the dose of cisplatin. However, the prevalence was not increased in patients with pretreatment eGFR < 60 mL/min/1.73 m^2^ with a 33.6% reduction in the dose of cisplatin. The effectiveness and the safety of cisplatin dose reduction in patients with pretreatment eGFR < 60 mL/min/1.73 m^2^ has not been clearly elucidated because of the ethical difficulties surrounding conducting such a prospective study [12, 24]. Although the one-year overall survival of the patients treated with a reduced dose of cisplatin-based chemotherapy was significantly lower than that of those treated with the standard dose in the CURE study using the same patient series [13], the results of this study demonstrated the safety of cisplatin dose reduction for cisplatin-ineligible patients in preventing nephrotoxicity.

Even considering our study results, cisplatin-based chemotherapy is not always safe and is not recommended for all the CKD-S patients with a solitary-functioning kidney with pretreatment eGFR < 60 mL/min/1.73 m^2^. However, the threshold and method to determine cisplatin-eligibility for patients with marginal kidney function are still controversial. Previous studies have shown about two-thirds of discordance in three methods of GFR estimation [5]. From the current study results, cisplatin-based chemotherapies could be recommended at least in CKD-S patients with marginal kidney function, such as around 10% of patients who are categorized in pretreatment 24hCCr > 60 mL/min, as well as eGFR < 60 mL/min/1.73 m^2^, as shown in Fig. 1. For patients with pretreatment eGFR of 50–60 mL/min/1.73 m^2^, 15/16 (93.7%) and 23/26 (88.5%) patients received a standard dose of cisplatin in bilateral- and solitary-functioning kidney patients, respectively. The prevalence of nephrotoxicity by more than 30% impaired kidney function during all courses of chemotherapy was 2/16 (12.5%) and 3/24 (12.5%), respectively.

There are several considerable limitations in this study. First, this study is a retrospective evaluation, and chemotherapy regimens or dose reductions for each cisplatin-ineligible patient were carefully selected under the discretion at each institute before initiation of the treatments. Patients with lower kidney function, higher age, and/or lower performance status received alternative regimens with or without platinum agents. In actuality, 90 of the 345 (26.1%) patients evaluated in the CURE study group, including 70 cisplatin-ineligible patients, did not receive cisplatin-based chemotherapies and of them, 57 received other platinum-based combination chemotherapy regimens consisting of carboplatin or nedaplatin, while 33 received chemotherapy without platinum agents. Second, only cisplatin but not methotrexate was considered as the nephrotoxic agent in this study. Third, the use of a detailed objective scoring system of medical comorbidities, such as the Charlson Comorbidity Index, was not evaluated in this study. Lastly, not only patients with nephroureterectomy but also patients who had unilateral hydronephrosis or who underwent a relief of the upper urinary tract obstruction were included in the group of patients with a solitary kidney. As such, variations in functional status of the urinary tract in patients with a solitary kidney are also a considerable limitation of this study.

## Conclusions

The results suggest that cisplatin-based chemotherapies may have more nephrotoxicity in patients with bilateral-functioning kidneys than in those with a solitary-functioning kidney. The nephrotoxicity of the chemotherapy may be of increased concern in CKD-M patients with bilateral-functioning kidneys than in CKD-S patients with a solitary-functioning kidney.

## Additional files

Additional file 1: **Figure S1.** Comparison of the number of patients with cisplatin dose-reduction between the four groups during four courses of cisplatin-based chemotherapy. A: First course, B: Second course, C: Third course, D: Fourth course. (PDF 104 kb)

Additional file 2: **Figure S2.** Comparison of the number of patients with nephrotoxicity between the four groups during four courses of cisplatin-based chemotherapy. A: Post first course, B: Post second course, C: Post third course, D: Post fourth course. (PDF 102 kb)

## Abbreviations

24hCCr: 24 h creatinine clearance
ANOVA: One-way repeated measures analysis of variance
C-G: Cockcroft–Gault formula
CKD-M: Medically-induced chronic kidney disease
CKD: Chronic kidney disease
CKD-S: Surgically-induced chronic kidney disease
eGFR: Estimated glomerular filtration rate
GC: Gemcitabine and cisplatin
GCD: Gemcitabine, cisplatin, and docetaxel
GFR: Glomerular filtration rate
MDRD: Modification of diet in renal disease
MEC: Methotrexate, epirubicin, and cisplatin
MVAC: Methotrexate, vinblastine, doxorubicin, and cisplatin
UC: Urothelial carcinoma
UTUC: Upper tract urothelial carcinoma
