# Supplementary figures and images for: A comparison of nephrotoxicity between patients with a solitary-functioning kidney and those with bilateral-functioning kidneys in cisplatin-based chemotherapy for advanced urothelial carcinoma: a Japanese retrospective multi-institutional study
Source: BMC Cancer. 2018 Mar 14;18:290. doi: 10.1186/s12885-018-4186-z (PMC5853031; doi:10.1186/s12885-018-4186-z)

# Supplementary figure 1

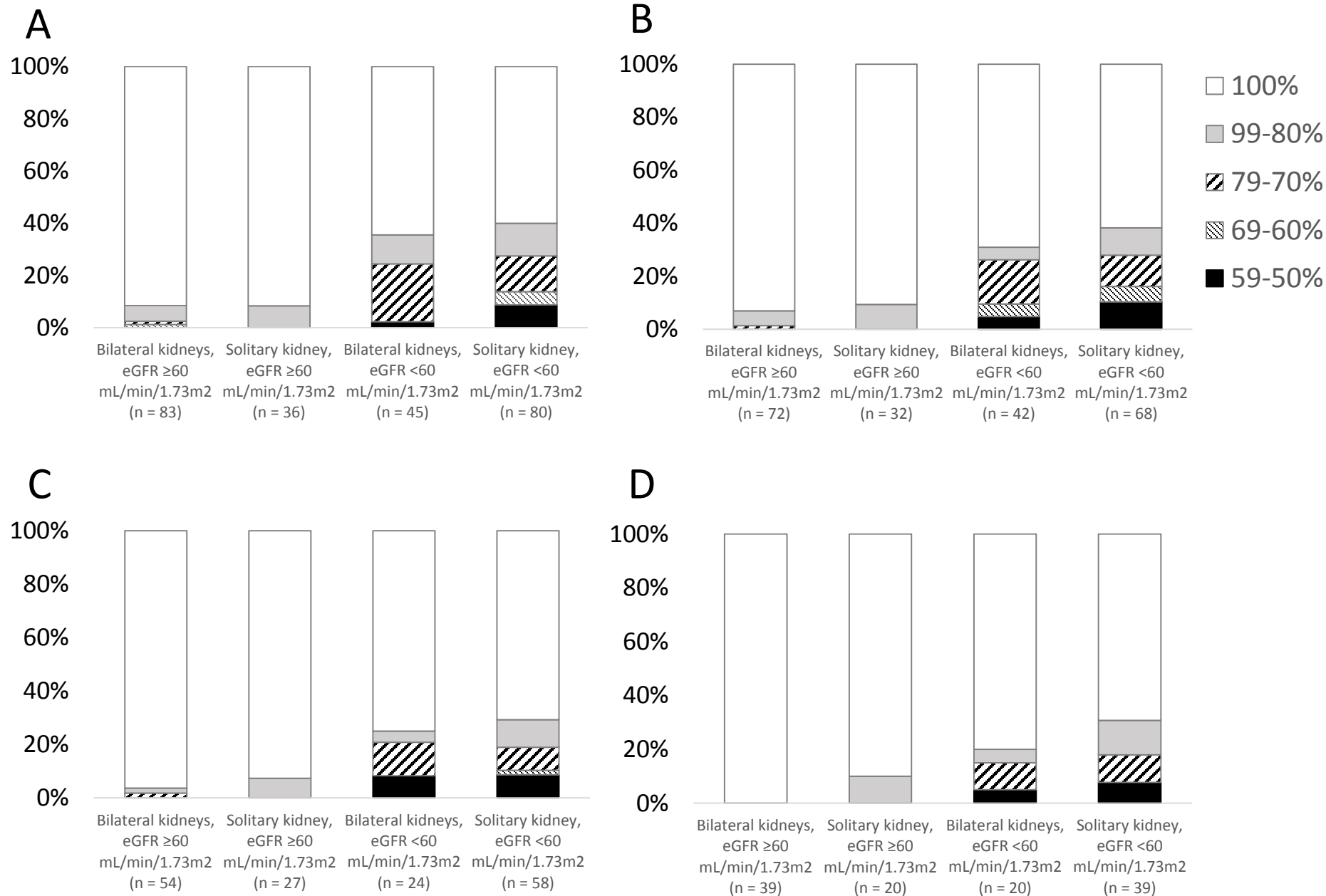

Supplement: Supplementary file 1 — Figure S1. Comparison of the number of patients with cisplatin dose-reduction between the four groups during four courses of cisplatin-based chemotherapy. A: First course, B: Second course, C: Third course, D: Fourth course. (PDF 104 kb) [file 12885_2018_4186_MOESM1_ESM.pdf]

## Supplementary figure 2

**A**

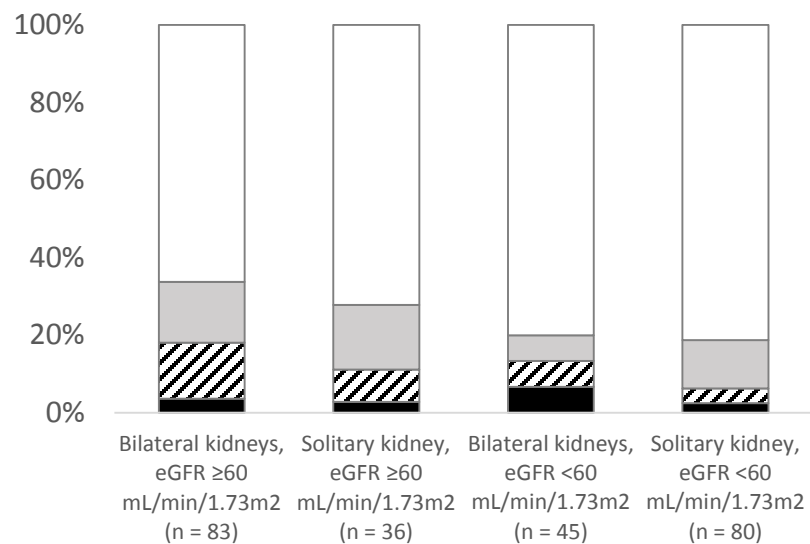

**B**

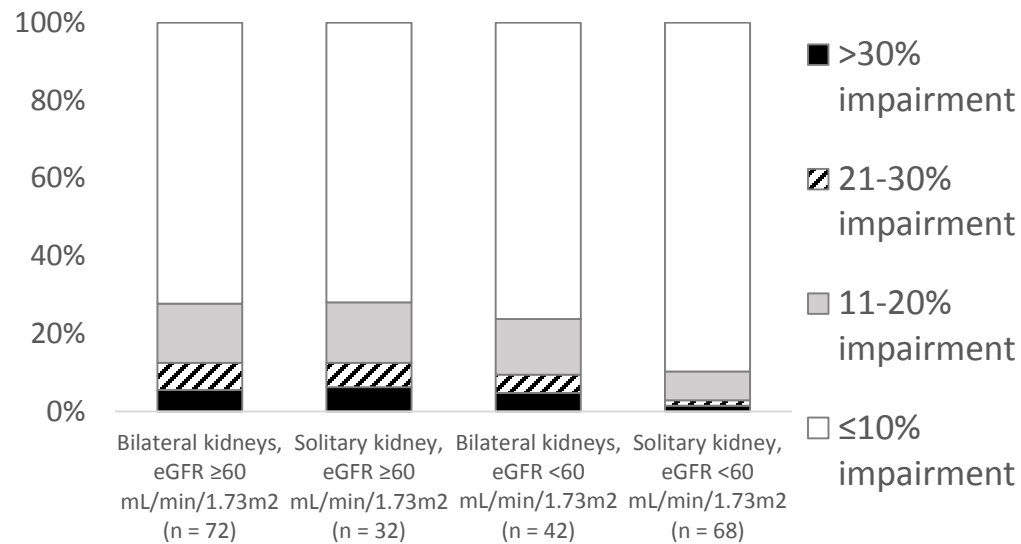

**C**

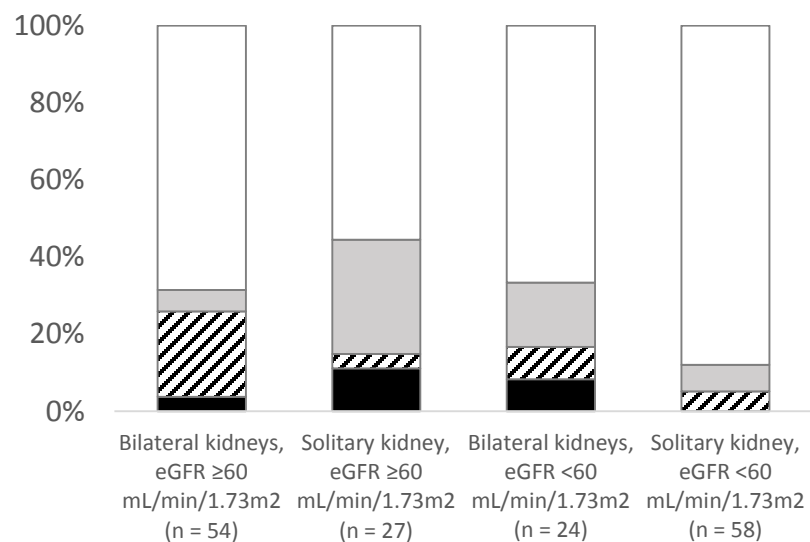

**D**

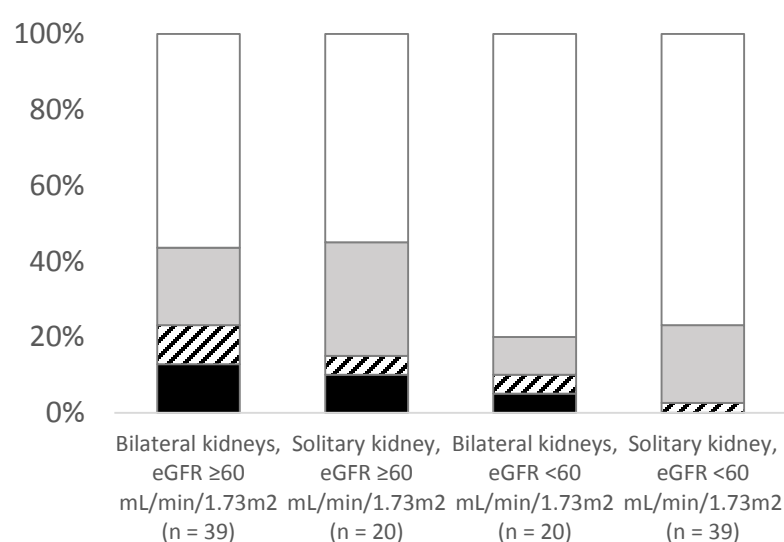

Supplement: Supplementary file 2 — Figure S2. Comparison of the number of patients with nephrotoxicity between the four groups during four courses of cisplatin-based chemotherapy. A: Post first course, B: Post second course, C: Post third course, D: Post fourth course. (PDF 102 kb) [file 12885_2018_4186_MOESM2_ESM.pdf]
